# Dishwashers as an Extreme Environment of Potentially Pathogenic Yeast Species

**DOI:** 10.3390/pathogens10040446

**Published:** 2021-04-08

**Authors:** Kamila Kulesza, Anna Biedunkiewicz, Karolina Nowacka, Maria Dynowska, Monika Urbaniak, Łukasz Stępień

**Affiliations:** 1 Department of Microbiology and Mycology, Faculty of Biology and Biotechnology, University of Warmia and Mazury in Olsztyn, Oczapowskiego 1A, 10-719 Olsztyn, Poland; alibi@uwm.edu.pl (A.B.); karolina.nowacka@uwm.edu.pl (K.N.); dynow@uwm.edu.pl (M.D.); 2 Plant-Pathogen Interaction Team, Department of Pathogen Genetics and Plant Resistance, Institute of Plant Genetics of the Polish Academy of Sciences, Strzeszyńska 34, 60-479 Poznań, Poland; lste@igr.poznan.pl

**Keywords:** fungi, microfungi, dishwashers, potential pathogens

## Abstract

The study aimed to compare the yeast species diversity in the specific environment of dishwashers, taking into account the potential risk for users. Yeasts were isolated from ten dishwashers and from tap water supplied to the appliances. Samples were collected for mycological analyses at the beginning of each month, from February to May 2016. Four dishwasher sites (rubber seals, detergent dispensers, sprinklers, and water drains) were analyzed. The microfungi were identified by the standard procedures applied in mycological diagnostics. To confirm species identification, molecular analysis was performed based on the sequences of the D1/D2 region. The presence of microfungi was detected in 70% of the investigated appliances. Rubber seals, detergent dispensers, and water drains were the most frequently colonized elements. Thirty-five yeast strains were isolated in this study, of which twenty-seven were obtained from dishwashers and eight from tap water. The strains belonged to six genera and six species (*Candida parapsilosis, Clavispora lusitaniae, Dipodascus capitatus, Exophiala dermatitidis, Meyerozyma guilliermondii*, and *Rhodotorula mucilaginosa*). Most of the strains came from rubber seals. In this way, it was demonstrated that the dishwashers’ condition is sufficient as an ecological niche for microfungi.

## 1. Introduction

Nowadays, more attention is being paid to the presence of potentially pathogenic microfungi indoors, whose diversity and strength may differ significantly from those in natural environments [1]. They may negatively affect the quality of life and health of room users, primarily through the allergenicity of spores, mycelium, and the toxicity of secondary metabolites [2]. Some opportunistic yeast species are the source of numerous skin and organ mycoses in humans with disturbance of the general biological balance [3].

Currently, we use many home appliances that facilitate the functioning of humans every day. This creates new alternative habitats for organisms capable of surviving in conditions considered extremely difficult [4]—for example, in appliances such as washing machines and dishwashers, representing an extreme environment for opportunistic microorganisms, including yeasts [5,6].

Specific and constantly fluctuating conditions prevailing in dishwashers, i.e., alternating high (60–80 °C) and low temperatures, variable pH (6.5–12), temporary dehydration, the high organic loads and concentration of NaCl, and mechanical stress caused by water sprinklers, allow for the growth and development of only selected microfungal species [7]. Novak-Babič et al. [6], during their study on the microbiological purity of washing machines, showed several species of the genus *Exophiala* (without *E. dermatitidis*) and *Candida parapsilosis, Rhodotorula mucilaginosa, Cystobasidium slooffiae, Meyerozyma guilliermondii*, and *Naganishia diffluens*. In turn, Zupančič et al. [7] observed that the same yeast species appeared in several independent dishwashers studied in different geographic areas. The authors defined the species as “dishwasher mycobiota” that occurred most often in various appliances but rarely in natural environments. The most frequently isolated species from dishwashers were the following: *Exophiala dermatitidis* and *E. phaeomuriformis, Candida parapsilosis, Dipodascus capitatus, Rhodotorula mucilaginosa*, and *Meyerozyma guilliermondii.* Occasionally emerging species were *Candida inconspicua, Clavispora lusitaniae, Metschnikowia fructicola, Pichia kudriavzevii*, and *Saccharomyces cerevisiae* [4,5,7,8]. The yeasts isolated from dishwashers are mainly oligotrophic, polyextremotolerant, and often opportunistic human pathogens [4,9]. Research carried out on various kitchen surfaces, in kitchens with and without dishwashers, has shown that dishwashers can be a source of fungal contamination. Species isolated from dishwashers were also found in hot aerosols and in the wastewater discharged from the appliances [7]. The most specialized microorganisms have adapted to variable stress factors, to the use of synthetic materials as a source of carbon, and can produce biofilms on various surfaces [1,10].

Rubber, plastic, and metal components of dishwashers allow the formation and growth of mixed bacterial and fungal biofilms [11]. Biofilm production gives the microorganisms extremely tolerant qualities that are well beyond a single species’ abilities [5,7]. A biofilm surrounded by extracellular polymeric substances protects against adverse environmental conditions, mechanical damage [12], and hygiene and antimicrobial substances, including antifungal agents [13]. The possibility of producing a biofilm is an important factor influencing the virulence of microorganisms toward a human. Due to this ability, microorganisms become resistant to the host’s immune system [14]. Microfungi are adaptive and pose a sanitary and epidemiological hazard to dishwasher users [4]. The fungi inside the dishwashers can potentially get into indoor areas with bioaerosols, on the user’s hands, or through washed dishes. In this context, dishwashers can be a potential source of various type of infections, which may lead to the development of fungal etiology diseases [7].

In this study, the species diversity of microfungi was checked on four elements of dishwashers (rubber seals, detergent dispensers, sprinklers, and water drains) and the water supplied to these appliances. The influence of using and applying various detergents on the occurrence of yeast in individual dishwashers was also analyzed.

## 2. Results

**Figure 1:**
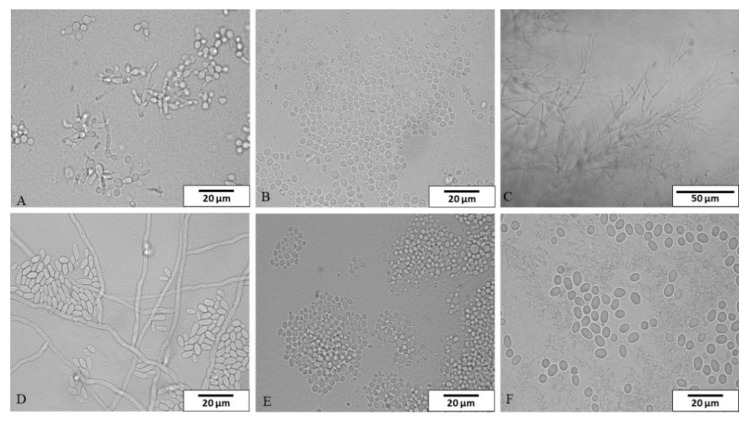
The species isolated from dishwashers and tap water in microcultures on Nickerson agar and examined under a light microscope (**A**) *Candida parapsilosis*, (**B**) *Clavispora lusitaniae*, (**C**) *Dipodascus capitatus*, (**D**) *Exophiala dermatitidis*, (**E**) *Meyerozyma guilliermondii*, (**F**) *Rhodotorula mucilaginosa*). Magnification: (**A**,**B**,**D**–**F**) 1000×, scale bar 20 μm; (**C**) 400×, scale bar 50 μm.

A total of thirty-five fungal strains were isolated in this study and identified using classical and molecular methods—twenty-seven from dishwashers and eight from tap water. The strains belonged to six genera and six species (Figure 1).

Altogether, 280 samples from different dishwasher sites (rubber seals, detergent dispensers, sprinklers, water drains) were collected in all research months: about 10% of the samples (27/280) were positive for fungi. Yeasts were found in seven out of ten dishwashers (70%). The most commonly contaminated elements of the dishwashers were rubber seals (4/10 appliances), detergent dispensers (4/10), and water drains (4/10). Only one sprinkler was colonized by yeast (1/10). Most of the strains were obtained from the dishwasher seals (14 isolates, 52%), followed by dishwasher dispensers (8 isolates, 29%) and sprinklers (4 isolates, 15%). One strain was found in the water drain (4%). The highest number of strains was obtained from dishwasher number 10 (8 isolates), slightly less from dishwashers 4, 5, and 6 (6, 5, and 3 isolates, respectively). Yeasts were not observed in the third and ninth dishwashers (Table 1). Dishwashers differed in the way of use (Table 2).

Most of the strains were isolated during the first collection in March (41%; 11/27 strains), less during the fourth in June (26%; 7/27 strains), third in May (18%; 5/27 strains), and the least during the second in April (15%; 4/27 strains). The differences between the number of strains in February and March were statistically significant (*p* = 0.0117) (Table 1).

The most common species was *Candida parapsilosis* (19 isolates), followed by *Exophiala dermatitidis* (5 isolates). *C. parapsilosis* most often appeared on the seals (60% of appliances; 9 strains) and less often in the dishwasher dispensers (40% of appliances; 8 strains) and sprinklers (20% of appliances; 2 strains). *E. dermatitidis* was present on seals (10% of appliances; 3 strains), sprinklers (10% of appliances, 1 strain), and in water drains (10% of appliances; 1 strain). The species *Clavispora lusitaniae* (rubber seal)*, Dipodascus capitatus* (sprinkler), and *Meyerozyma guilliermondii* (rubber seal) were recorded only once. Of the isolated yeast species, three were classified as biological safety level (BSL)-2, while 3 were BSL-1 (Table 1 and Table 3). No statistically significant differences were found between the dishwashers’ parts and their yeast counts (*p* < 0.05).

**Figure 2:**
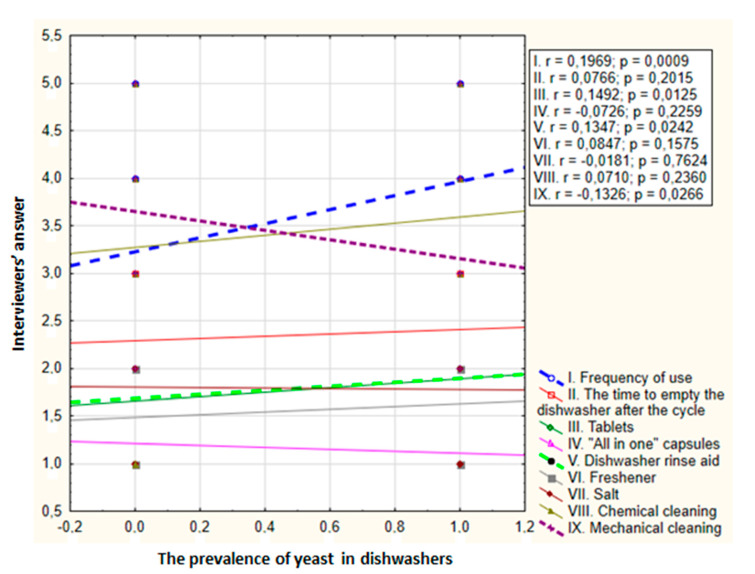
Relationship between dishwasher yeast counts and parameters related to the use of dishwasher in ten appliances over four months (r—Spearman coefficient; p—significance level; dashed line—statistically significant differences). Possible answers in the survey: **I**. frequency of use—1. less often than once a week, 2. once a week, 3. several times a week, 4. once a day, 5. several times a day; **II**. time to empty the dishwasher after the cycle—1. a long time after completion, 2. a few hours after completion, 3. immediately after completion; **III**–**VII**. chemicals used—1. no, 2. yes; **VIII**. chemical cleaning; **IX**. mechanical cleaning—1. less often than once a year, 2. once a year, 3. twice a year, 4. quarterly, 5. once a month.

Based on the survey, some of the dishwasher operation parameters were analyzed for the incidence of microfungi. A positive correlation was found between the frequency of using the dishwasher and isolated yeast counts (correlation coefficient 0.1969, *p* = 0.0009). The more often the dishwasher was used, the more yeast it contained. A negative correlation was observed between the frequency of mechanical cleaning and the number of yeasts (correlation coefficient −0.1326, *p* = 0.0266). The less frequently the dishwasher was cleaned, the more yeast was present in it. There were no statistically significant differences between the number of isolated yeast species and the use of cleaning supplies, except for dishwasher rinse aid, and a positive correlation was found (correlation coefficient 0.1347, *p* = 0.0242). Using a rinse aid increases the species diversity of fungi in the appliances (Figure 2).

**Figure 3:**
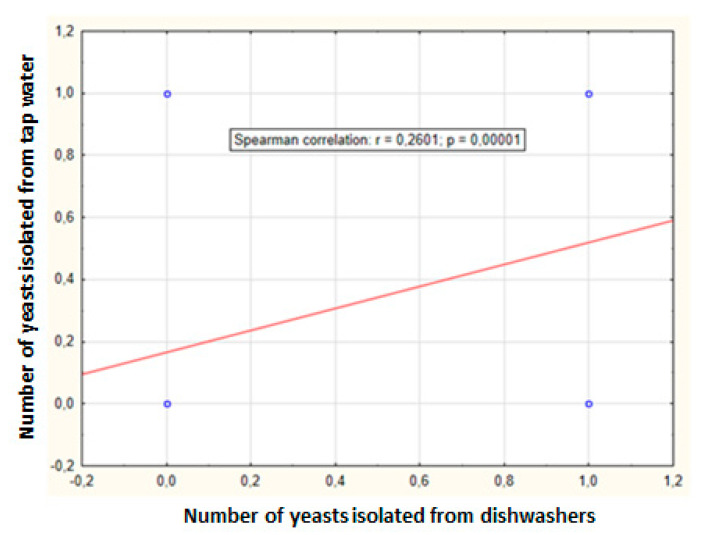
Relationship between dishwasher and tap water yeast counts obtained in ten appliances over four months (r—Spearman coefficient, p—significance level).

Tap water samples were also collected from the systems supplying the dishwashers to evaluate possible yeast contamination. In the simultaneously tested water, eight isolates were found in 28 samples (29%): four species from four genera. Again, *Candida parapsilosis* was the most common species in the tap water supplied to dishwashers (four times). *Meyerozyma guilliermondii* was found less frequently (two times), while *Clavispora lusitaniae* and *Rhodotorula mucilaginosa* were isolated only once. *Rhodotorula mucilaginosa* has only been found in tap water. One isolated species was assigned to BSL-2 and three species to BSL-1. Positive correlation was found between the number of yeasts isolated from tap water and the dishwashers (correlation coefficient 0.2601, *p* = 0.00001) (Figure 3).

## 3. Discussion

Nowadays, people spend most of their time indoors. Therefore, they are exposed to microorganisms’ adverse effects from water and air [15]. Therefore, more attention is paid to microbe presence inside buildings, whose standards are continually being raised. Unconsciously, humans contribute to selecting microorganisms forced to adapt to unnatural substrates and habitats with specific physicochemical parameters and tolerance to the chemicals used [16]. 

Humans have a wide array of host defenses against fungal infections. The most severe mycoses occur in people with defects in these mechanisms. The host’s first line of defense consists of barriers, such as skin and mucous membrane. The most effective defense mechanism is the proper functioning of the immune system [17]. As a result of the currently used treatments, many diseases do not pose a threat to people, and life expectancy is increased. However, the number of immunocompromised patients has increased significantly in recent years. People with immunodeficiency are at risk of opportunistic infections, especially those caused by fungi [18]. The increasing use of biological agents in treating various diseases, including cancer, autoimmune, bacterial, and fungal diseases, caused an increased risk of opportunistic infections due to host immunity disorders [19]. Until recently, organic weakness or an immunocompromised host was thought to be the only predisposing factor to opportunistic fungal infections. However, yeasts are actively involved in pathogenesis, using aggression mechanisms called virulence factors [20]. These factors include adhesion capacity, biofilm formation, production of exopolysaccharides, and fungal dimorphic nature [21]. Only a few pathogens can infect healthy people. The traits responsible for the virulence of microorganisms have been associated with adapting these species to adverse environmental conditions. Fungal infection in humans is somewhat random, it has no role in the evolution of fungi, and therefore it is typically opportunistic [22].

The vast majority of microfungi are characterized by extraordinary ecophysiological plasticity. They can occupy ecological niches often inaccessible to other decomposers. Human-made environments, particularly within dwellings, create unnatural habitats for a limited number of fungal species whose natural niches have not been sufficiently studied [23]. Polyextremotolerant fungi are characterized by great adaptability to inhabit new ecological niches related to these significant fungal tolerances to stress [1,23]. An example may be thermotolerance (development at temperatures above 37 °C). It is an important feature in the pathogenesis of specialized fungal species, resulting from increased ambient temperature [1]. Fungal tolerance to extreme environmental conditions and their adaptive abilities have been associated with their pathogenicity toward humans, and therefore indoor species may be opportunistic pathogens [24]. The natural environment of the opportunistic fungal pathogens is not the hosts but their surroundings [5]. A previous report suggests that changes in the environment affect the interactions between microorganisms and humans unexpectedly and dangerously. Thus, dwellings may pose a potential risk to their users [2].

Constant access to water in dwellings creates habitats with increased humidity, which is necessary for developing microorganisms. Due to the increased humidity and temperature and the presence of natural and unnatural carbon sources, kitchens and bathrooms are exposed to the presence of microorganisms [7]. They create new ecological niches for numerous human pathogens [9,25]. Kitchen drains and dish dryers are often colonized by black yeast species such as *Exophiala dermatitidis, E. phaeomuriformis*, and *Aureobasidium melanogenum*. The species *C. parapsilosis* is the most commonly recorded white yeast [7]. On the other hand, shower curtains and floors were often inhabited by yeasts of the genus *Candida*, *Cryptococcus, Rhodotorula,* and black yeasts of the genera *Aureobasidium* and *Exophiala* [9,25].

Home appliances, which also provide a new habitat for potentially pathogenic microorganisms, have become widespread. In recent years, attention was drawn to the diversity of microorganism species in household appliances, such as washing machines [6,26] or dishwashers [4,5,7,8]. In laundry and dishwasher conditions, only microorganisms with polyextremotolerant properties can survive and grow [11]. The growth, proliferation, and other physiological properties of microorganisms are closely related to the biofilm formation process. 

A biofilm is defined as a functional, phylogenetically diverse community of bacteria, fungi, and algae that adheres permanently to a surface [27]. Biofilm production ensures increased cell viability and nutrient availability. It also protects the microorganism from the harmful effects of toxic substances produced to increase the competition between the species [28]. It also precipitates the use of polymers as a substrate and medium. Although synthetic compounds do not occur in nature, they can be a suitable material for the development of microfungi, which can degrade synthetic polymers [29] by destroying ester bonds. As a result, they can adhere to the surface and colonize it [30]. Any type of synthetic material inhabited by microorganisms in contact with water, sunlight, or oxidants can be subject to weathering and degradation [31]. Fungi can grow on polyacrylamide, silicone [32], and even on glass materials [33]. Nutrients can come from outside of the appliances or arise from the degradation of polymers of technical materials due to the microorganism’s ability to produce free radicals or enzymes [34]. After decomposing various substances, including organic and inorganic contaminations (dirt and detergents), microorganisms produce volatile organic compounds, mainly dimethyl disulfide, resulting in an unpleasant odor in commonly used appliances [35]. Chemical and physical methods, i.e., high temperature, UV, X-ray, gamma radiation, and elevated pressure, protect synthetic materials from damaging effects and colonization by microorganisms. Disinfection measures are often used, but their abuse may not have the desired effect and negatively affect human health and the environment. With the long-term use of detergents, it is possible to select resistant strains dangerous to humans [36].

In this study, the presence of five yeast species from five genera was found in 70% of dishwashers, regardless of the place of sampling. The presence of microfungi has also been demonstrated in 40% of the water sprinklers (three species from three genera), in 40% of detergent dispensers (one species, *C. parapsilosis*), and 10% of sprinklers (one species, *E. dermatitidis*). These results correspond to the data obtained by Zupančič et al. [7]. These authors isolated microfungi from 43% of drains, 13% of detergent dispensers, and 7% of water sprinklers. In the fungal biofilm community on rubber seals in dishwashers researched by Raghupathi et al. [11], Ascomycota dominated, followed by Basidiomycota. Of the Ascomycota subclass, the genera *Candida*, *Debaryomyces,* and *Saccharomyces* were present in all appliances, while black yeasts (genera *Aureobasidium* and *Exophiala*) were reported in 33% of dishwashers. The genera *Rhodotorula* (in 90% appliances) and *Cryptococcus* (86% appliances) have been reported from the Basidiomycota subclass. In turn, the genera *Wallemia* and *Trichosporon* were found in more than 50% of dishwashers. These results correspond to those of Zupančič et al. [7], who isolated black yeast, *Exophiala dermatitidis* and *E. phaeomuriformis;* white yeast, *Candida parapsilosis;* and pink yeast *Rhodotorula mucilaginosa,* from mixed biofilm communities. All of them are opportunistic human pathogens and can be a potential source of fungal contamination in rooms.

Most foregoing research focused on microfungi presence on the rubber seals in dishwashers [4,5,7]. Pioneering research into dishwashers was done by Zalar et al. [4] and has proven that microfungi prevalence on seals was approximately 62%. In our research, microfungi were present on 60% (four species) of the rubber seals of the controlled appliances. Zupančič et al. [7] also checked other parts (doors, interior side walls, detergent dispensers, rinse-aid dispensers, cutlery racks, side nozzles, and sprinklers) and the water supplied to the dishwashers. They reported 83% of the controlled appliances were colonized by microfungi (29 species from 10 genera). From 189 tested dishwashers, 120 strains of fungi, classified into 14 genera, were obtained. In turn, Dögen et al. [5] showed the presence of fungi on seals in 17.7% of dishwashers, Gümral et al. [8] in 24.5%, and Zupančič et al. [7] in 57% of the tested seals. 

The most common dishwasher colonizers in previous research were opportunistic black yeasts of the genus *Exophiala*, namely *E. dermatitidis* and *E. phaeomuriformis*, which were present in 30–50% of dishwashers. In our research, only *E. dermatitidis* was isolated in 20% of appliances. The common occurrence of *Candida* species, mainly *C. parapsilosis*, has also been noted. In our research, this species was present in 70% of the tested dishwashers. Other yeasts documented in dishwashers were *Dipodascus capitatus, Rhodotorula mucilaginosa,* and *Meyerozyma guilliermondii*. All these species colonized dishwashers controlled in our research, except *Rhodotorula mucilaginosa*. Other species appeared in dishwashers sporadically [4,5,7,8].

*C. parapsilosis* was the dominant species in the researched appliances. This species was present in tap water and all elements of dishwashers, except for the water supply. Other authors often noted *C. parapsilosis* in dishwashers [4,5,7], washing machines [6], and tap water [3]. *C. parapsilosis* was a frequent colonizer of kitchens without dishwashers. This species was isolated from drains, kitchen sinks, countertops, and dish dryers [37]. The ecology of *C. parapsilosis* is poorly understood. The research of Döğen et al. [38] showed that almost all isolates of *C. parapsilosis* from laundry machines could grow at temperatures from 10 to 37 °C, displayed halotolerance (NaCl ≥ 10%), and alkalitolerance (pH > 10), which shows the adaptability of this species to extreme conditions. *C. parapsilosis* was recovered from a variety of sources and frequently isolated from clinical specimens of all sorts. Next to *C. tropicalis* and *C. glabrata*, it is the most common cause of opportunistic infections, after *C. albicans* [39]. Although *C. parapsilosis* is a human commensal, it can also be an opportunistic pathogen associated with nosocomial infections, and its incidence is increasing worldwide [38]. This species is emerging as an important infection source in neonates, patients receiving parenteral nutrition, transplant recipients, and patients undergoing corneal surgery [40,41].

Particular attention should be paid to the *Exophiala* genus fungi, which often belong to BSL-2 (potential pathogens) [39]. These fungi have a dimorphic character so that they can pass from the yeast-like to the hyphae state. Moreover, melanin in the cell wall can protect fungal cells against lysis, phagocytosis, different kind of environmental conditions, and stress, as well as the action of antifungal agents [42]. Therefore, black yeasts can cause opportunistic infections of varying intensity [43,44]. *Exophiala* species can break down the aromatic hydrocarbons, assimilate detergents [25], survive at high temperatures and pH [4], and also tolerate cycloheximide [38]. Due to their properties, they are often recorded in habitats with a high humidity degree [8]. Frequent colonization of rubber seals by *E. dermatitidis* and *E. phaeomuriformis* species has been shown, which were detected in 15–47% of dishwashers [4,5,7]. In our research, *E. dermatitidis* was present in 20% of the tested appliances on all controlled dishwasher parts, except for the dishwasher dispenser. The presence of the species *E. phaeomuriformis* was not found, while other authors noted this species on rubber seals, side nozzles, and dishwasher sprinklers [4,5,7]. The lower occurrence of *E. phaeomuriformis* in dishwashers, which temporarily reached high temperatures may be related to the lower maximum growth temperature of this species (38 °C) compared to that of *E. dermatitidis* (42 °C). Thus, the colonization by *E. phaeomuriformis* occurs at lower ambient temperature [45]. *E. dermatitidis* has also been demonstrated in kitchens with dishwashers, which may indicate the contamination of indoor spaces through domestic appliances. In kitchens without dishwashers, greater species diversity of the genus *Exophiala* was observed [7]. 

Research indicates that among the genus *Exophiala*, the species *E. dermatitidis* is the most virulent and can cause infections of the greatest severities in humans [46]. *E. dermatitidis* produces exopolysaccharides, responsible for its thermotolerance and biofilm formation, drug resistance, and avoiding host resistance, which is closely related to its virulence [47]. It is a polyextremophilic species, metabolically active in a wide range of pH (2.5–12.5) and temperature (4–40 °C) [1,4], making it able to survive in unfavorable conditions of dishwashers. *E. dermatitidis* can be the cause of various diseases, from cutaneous and subcutaneous infections to systemic, pulmonary, neurotropic, and gastrointestinal infection, particularly in immunocompromised patients [47], recently also bloodstream infections [48]. The species is rarely associated with the natural environment, and its occurrence increases in environments contaminated with aromatic hydrocarbons (cyclic or non-cyclic) [47]. It is widespread in home environments such as bathrooms [25], saunas [45], and dishwashers [4,5,7].

*Meyerozyma guilliermondii* is a species ubiquitous in nature and found in the dishwashers’ extreme environment. Its presence on appliance seals was demonstrated in the studies of Zalar et al. [4], while Zupančič et al. [7] documented this species on the side nozzle, doors, and dishwasher drain. In our research, the species was also present in tap water and on rubber seals. *M. guilliermondii* is considered an opportunistic human pathogen and accounts for approximately 2% of all yeast clinical isolates [49,50].

Earlier research revealed a constant presence *Dipodascus capitatus* on seals [5], cutlery racks, rinse-aid dispensers, side nozzles, and water drains [7]. In our research, this species was recorded only in the water drain. *D. capitatus* can sometimes be isolated from the environment [51]. They can be found in the microbiota colonizing human skin and the digestive and respiratory tract mucosa [52]. This species is often noted as a human pathogen, mainly in the blood of immunocompromised patients, especially those with leukemia [53]. It occurs primarily in heated habitats [40] and is a BSL-2 pathogen [54]. Therefore, the frequent isolation of this species in dishwashers is threatening. Its repeated discovery in appliances indicate that the conditions inside them are suitable for its growth and development. Kitchen studies have shown that it does not spread inside indoor spaces [7]. 

*Clavispora lusitaniae* is another species classified as BSL-2 and isolated from dishwashers. This species has been reported by Zupančič et al. [7] once on the side nozzle, and in our research, it appeared on one rubber seal. The species is a part of normal mycobiota but can lead to the colonization of the urinary and digestive systems [40]. *C. lusitaniae* is regularly recovered in clinical specimens, mainly in cancer patients who received cytotoxic chemotherapy or bone marrow transplantation [55]. It is responsible for about 19.3% of all infections caused by non-*Candida albicans* species [56]. Its frequent occurrence in high-risk patients and a broad spectrum of resistance to antifungal drugs, including amphotericin B, indicates the need for research on this species [57].

Water is a potential contamination source of dwellings and household appliances [24]. Four yeast species were recorded in tap water, of which three were also isolated from the dishwashers. The presence of the same yeast species in tap water supplied to the appliances and in dishwashers and the positive correlation between the number of yeasts isolated from tap water and dishwashers confirms the conclusion of other authors [7,58] on the possibility of fungal transmission to appliances from tap water. However, only some yeasts, adapted to the extreme condition in dishwashers, can survive. *Rhodotorula mucilaginosa* is a ubiquitous species and is closely related to various aquatic environments [40]. It has been found by Zupančič et al. [7] both in tap water and on the seal and door of dishwashers. Zalar et al. [4] showed this yeast on seals. In our research, it was not present in appliances but only in the supplied water. *R. mucilaginosa* is probably the only species of the genus *Rhodotorula* that causes human infections. It has been found that this species can induce fungemia, endocarditis in immunocompromised hosts, and meningitis [59].

During the dishwasher work, various physical and chemical factors simultaneously affect the microorganism community. Fungi in dishwashers are exposed to constant environmental pressure. Therefore, determining the influence of a specific parameter on the microfungi survival and development is complicated. The relationship between temperature (the only variable factor during the washing cycle) and the presence of microorganisms in dishwashers was shown by Brandt et al. [60] and Brandt and Dirk [61]. As the temperature increased, yeast counts decreased, indicating an adverse effect of energy-saving measures on appliance hygiene. Moreover, Brandt and Dirk [61] observed that the increased microorganism in dishwashers did not affect the dishes’ microbiological cleanliness and hygiene, which is probably related to the drying process. Humidity is an important parameter influencing the growth and development of fungi. Zupančič et al. [7] showed greater species diversity in hydrated biofilm samples than in dehydrated biofilm samples. This observation may indicate that increased humidity in dishwashers (resulting from an improper drying process or closing the dishwasher door without airing it out) may adversely affect the hygiene of the dishwasher. These studies also showed that hot aerosols, released by opening dishwashers before the cooling process is complete, contribute to the spread of fungi in the kitchens.

During our research, due to the small number of samples, statistically significant differences were found only between the parameters of use, which had the greatest variety of responses from interviewers. A statistically significant correlation was found between the frequent use of the dishwasher and microfungi counts. Perhaps it is related to the regular organic matter supply and higher humidity inside the appliances. Pre-rinsing the dishes with food scraps before placing them in the dishwasher could reduce the occurrence of microfungi. Statistically significant differences were also observed between the frequency of mechanical cleaning and the reduction of yeast counts. Therefore, it seems that regular cleaning of the appliances is important for reducing the microfungal contamination. There was also a correlation between the use of rinse aid and increased yeast species diversity. No statistically significant differences were found between emptying the dishwasher after the washing cycle, chemical cleaning, using a capsule, freshener, and salt and the dishwashers’ microbiome. In this study, no correlation was found between some physicochemical parameters and the variety of yeast in dishwashers, which does not mean that none of these correlations existed.

## 4. Materials and Methods

### 4.1. Fungal Material

Thirty-five yeast strains isolated in this study have been deposited in the fungi collection of the Department of Microbiology and Mycology, University of Warmia and Mazury in Olsztyn, Poland.

### 4.2. Collection of the Fungal Samples

The study material was collected from 10 appliances located in kitchens in two cities of north-eastern Poland (Olsztyn, Ostrołęka, Poland). Mycological analyses of samples from dishwashers and tap water supplied to them were carried out at the beginning of each month, from February to May 2016. Dishwashers were different in the frequency of use, emptying of dishwashers after the end of the washing cycle, the type of detergents used, and the cleaning frequency (chemical and mechanical). All dishwashers had been in use for more than six years. All users had used dishwasher tablets. All machine and usage parameters were assessed by means of a questionnaire (Table 2).

Samples were collected using sterile cotton swabs by rubbing the dishwasher element’s surface (seals around the dishwasher’s doors, detergent dispensers, water sprinklers, and water drains) and next placed in sterile containers. In addition, tap water was collected from the kitchens, where the appliances were located, into sterile 1000 mL bottles. After transporting the samples to the laboratory of the Department of Microbiology and Mycology of the University of Warmia and Mazury in Olsztyn, within 24 h of collection (storage temperature 4 °C), swabs were placed in Sabouraud broth and then incubated at 37 °C for 24–72 h. Sabouraud’s broth was used to increase the likelihood of isolating the fungal species present on the swabs, as it gives a relatively equal chance for yeasts to grow at different growth rates. All cultures in which changes in the form of clump, sediment, and/or membrane on the broth surface were observed were considered positive and transferred using the “drop-plate” method onto Sabouraud agar with chloramphenicol (0.05%) to eliminate bacteria. Water samples were filtered through sterile membrane filters Ø 0.45 µm (MerckMillipore, Darmstadt, Germany) and placed on Sabouraud agar with an antibiotic (0.05% chloramphenicol). After incubation (37 °C/24–72 h), yeast colonies were passaged onto slants with Sabouraud agar. Gross identification of yeasts was performed according to commonly accepted procedures for mycological laboratories [59]. The growth colonies were evaluated for macroscopic features on Sabouraud agar with chloramphenicol (size, color, shape, surface structure, sheen, consistency) and microscopic features in microculture on Nickerson agar (size, shape, and arrangement of blastospores, presence of pseudomycelium, and chlamydospores) and biochemical features (zymograms— the ability to ferment sugars) [62]. The media used in the experiment are given in Table 4.

### 4.3. Identification of Yeasts

The identification of yeasts was based on the morphological (macro- and microscopic) and biochemical (zymograms) features and was compared to data based on sequence analyzes during molecular identification. 

#### 4.3.1. Morphological Identification of Yeasts

The yeasts were identified using specialized keys for species identification of fungi: de Hoog et al. [54], Kurtzman and Fell [64], and Kurtzman et al. [39]. Based on de Hoog et al. [54] and Kurtzman et al. [39], the biological safety level (BSL) was determined for yeast species.

#### 4.3.2. Molecular Identification of Yeasts

Purified yeast cultures were lyophilized and weighed. Genomic DNA extraction was performed using the Plant and Fungi DNA purification Kit (EURx, Gdansk, Poland), according to the manufacturer’s recommendation. The concentration of genomic DNA for each sample was measured using NanoDrop 1000 UV–Visible Spectrophotometer (Thermo Fisher Scientific, Waltham, MA, USA), and the DNA extracts were stored at −20 °C. Polymerase chain reactions (PCRs) were done in 50 µL aliquots using C-1000 thermal cyclers (Bio-Rad, Hercules, CA, USA). Each PCR was conducted using 1.25 U of Color Taq DNA polymerase (EURx, Gdansk, Poland), 5 µL of 10× Pol Buffer B, 0.2 mM of each dNTP, 0.5 µM of forward/reverse primers, and about 20 ng of yeast DNA. PCR conditions were followed: 5 min at 95 °C, 35 cycles of 1 min at 95 °C, 1 min at 56 °C (annealing), and 1 min at 72 °C, and the final extension 7 min at 72 °C. For the PCR amplification of a D1/D2 region of 26S rRNA gene, the primers NL1 (5′-GCATATCAATAAGCGGAGGAAAAG-3′) and NL4 (5′-GGTCCGTGTTTCAAGACGG-3′) [65] were used. Both yeasts [66] and filamentous fungi [67,68] were identified based on amplification and sequencing of the large subunit ribosomal RNA sequences (D1/D2 domains). Amplicons were separated in 1.5% agarose gel (EURx, Gdansk, Poland) with a SimplySafe nucleic acid stain (EURx, Gdansk, Poland). 

For sequence analysis, obtained amplicons were purified as described earlier by Kozłowska et al. [65]. Afterward, the purified amplicons were labeled using a reverse primer (NL4) and the BigDye Terminator 3.1 kit (Applied Biosystems, Foster City, CA, USA), according to Tomczyk et al. [69]. Sequences were analyzed using the BLASTn algorithm and compared with reference sequences from the GenBank database.

### 4.4. Statistical Analysis

All statistical analyses were performed using the STATISTICA13 software (StatSoft). All analyzed data were categorical (qualitative) variables. The statistical analyses were conducted at a 95% confidence level (significance level *p* < 0.05). The Friedman analysis of variance (ANOVA) was used to test the yeast counts isolated from dishwashers over four months. The Bonferroni test, following a significant Friedman ANOVA result, was used to identify at which months the differences in yeast counts were observed. Following a significant Friedman ANOVA result, the non-parametric Wilcoxon matched-pairs test was used to determine the significance of changes each month for the number of yeasts isolated from dishwashers. Differences among the parts of the dishwasher in yeast counts were tested with non-parametric Kruskal–Wallis tests. A scatterplot of dishwasher yeast counts and parameters related to the dishwasher, dishwasher yeast counts, and water yeast counts was drawn. The strength of this correlation was measured with the Spearman correlation coefficient.

## 5. Conclusions

The conducted research confirms that yeasts are euryecological organisms with exceptional adaptability and resistance to environmental factor fluctuations, using a broad spectrum of natural and human-made ecological niches for growth and reproduction. One of them is dishwashers, which can have a stimulating or limiting effect on microbes’ growth. This is particularly important in the case of potentially pathogenic microorganisms that can pose a sanitary and epidemiological hazard. Despite abundant information about potential sources of contamination and microorganisms’ appearance in various dishwasher components, information on physicochemical factors reducing their occurrence is supplemented (e.g., the influence of high and low temperatures) but still incomplete. Due to the changing ecological trends (preferring lower temperatures, lower water consumption, new dishwashers with a container for reuse of water from the final rinse, and a reduced amount of detergents during washing cycles), it is necessary to further evaluate the impact of environmentally friendly measures in dishwashers on the occurrence of microfungi, especially yeasts. Natural antifungal agents’ effect on the frequency and diversity of fungi isolated from dishwashers should also be studied. Therefore, further research is needed to investigate the effect of ecological parameters (biological and physicochemical) on microorganisms living in extreme conditions. Due to the small number of samples (10 dishwashers tested four times), the conducted research is purely qualitative. The obtained results indicate the probability of a relationship between the frequent use of the dishwasher and rinse aid on the increase of the fungal species diversity and between frequent mechanical cleaning and the reduction in yeast counts. 

Based on our own and other authors’ research, it is assumed that some activities during the use of dishwashers may limit the spread of microorganisms in the dwellings. It may be suggested that the use of high temperature during the cycle, the correct drying process, not opening the dishwasher immediately after the cycle is completed, airing the dishwasher after it has cooled down, rinsing the dishes before placing them in the appliance, and regular mechanical cleaning may have a positive effect on the microbiological hygiene of dishwashers.

## Figures and Tables

**Table 1:** List of isolated species from tap water and researched elements of dishwashers, taking into account the biosafety level (BSL) (1–10, dishwasher number; I—March, II—April, III—May, IV—June, research seasons).

Species	BSL	Rubber Door Seals	Sprinklers	Water Drain	Dishwasher Dispensers	Tap Water
*Candida parapsilosis*	1	I-4, 6, 7, 8, 10 II-4, 5, 10 IV-4	III-10IV-2		I-4, 5, 10 II-10 III-10 IV-1, 4, 10	I-5 II-10 III-10 IV-5
*Clavispora lusitaniae*	2	III-8				II-8
*Dipodascus capitatus*	2		III-4			
*Exophiala dermatitidis*	2	I-5 III-5 IV-5	I- 6	I-6		
*Meyerozyma guilliermondii*	1	IV-10				I-10 IV-10
*Rhodotorula mucilaginosa*	1					I-4

**Table 2:** Parameters related to the use of dishwashers (1–10).

Parameters of Use	1	2	3	4	5	6	7	8	9	10
Frequency of use	A few times a week	Once a day	Once a day	A few times a week	Once a day	Once a day	Once a week	A few times a week	Sporadically	Several times a day
Time to empty the dishwasher after the cycle	A few hours after the end of the cycle	A few hours after the end of the cycle	Immediately after the cycle	Immediately after the cycle	Immediately after the cycle	A few hours after the end of the cycle	A few hours after the end of the cycle	A few hours after the end of the cycle	A few hours after the end of the cycle	A few hours after the end of the cycle
Chemical cleaning	Once a month	Twice a year	Rarely	Twice a year	Once a month	Once per quarter	Once per quarter	Once per quarter	Rarely	Twice a year
Mechanical cleaning	Once per quarter	Twice a year	Twice a year	Once a month	Twice a year	Once a year	Once a month	Once per quarter	Once a month	Twice a year
Use of “all-in-one” capsules	+						+	+		
Use of dishwasher rinse aid	+			+	+	+	+		+	+
Use of freshener	+	+		+			+			+
Use of salt	+	+			+	+	+	+	+	+
Number of yeasts isolated from dishwashers	1	1	0	6	5	3	1	2	0	8
Number of yeasts isolated from tap water				1	2			1		4

**Table 3:** Identification of fungal strains based on the sequence of the D1/D2 region of the large ribosomal subunit in comparison with reference sequences from GenBank Database (Designation of fungal strain: 1–35, number of isolate; TW—tap water, RS—rubber seals, S—sprinklers, WD—water drain, DD—dishwasher dispenser; 1–10, dishwasher number; e.g., 07.02.16—sampling date).

Designation of Fungal Strain	Identified Fungal Species	Sequence Identity
1/TW/4/07.03.16	*Rhodotorula mucilaginosa*	99% identity to the *Rhodotorula mucilaginosa*, acc. numbers: JQ965876.1, KY109096.1, MT550663.1
2/TW/5/07.03.16	*Candida parapsilosis*	98% identity to the *Candida parapsilosis*, acc. numbers: MK110313.1, MH704191.1, MT001243.1
3/TW/10/07.03.16	*Meyerozyma guilliermondii*	99.5% identity to the *Meyerozyma guilliermondii*, acc. numbers: KX791359.1, KF268282.1, JQ277247.1
4/RS/4/07.03.16	*Candida parapsilosis*	100% identity to the *Candida parapsilosis*, acc. numbers: MT001266.1, MH545914.1, MK110314.1
5/RS/5/07.03.16	*Exophiala dermatitidis*	94% identity to the *Exophiala dermatitidis*, acc. numbers: KF928509.1, MT023625.1, MH878058.1
6/RS/6/07.03.16	*Candida parapsilosis*	100% identity to the *Candida parapsilosis*, acc. numbers: FJ480839.1, MT176532.1, KU316730.1
7/RS/7/07.03.16	*Candida parapsilosis*	100% identity to the *Candida parapsilosis*, acc. numbers: MT176532.1, MN067761.1, KU316730.1
8/RS/8/07.03.16	*Candida parapsilosis*	100% identity to the *Candida parapsilosis*, acc. numbers: LC415311.1, MT176532.1, MH481614.1
9/RS/10/07.03.16	*Candida parapsilosis*	100% identity to the *Candida parapsilosis*, acc. numbers: MK110312.1, MK110091.1, MT001266.1
10/S/6/07.03.16	*Exophiala dermatitidis*	98% identity to the *Exophiala dermatitidis*, acc. numbers: KT756672.1, AY731737.1, MH876925.1
11/WD/7/07.03.16	*Exophiala dermatitidis*	91% identity to the *Exophiala dermatitidis*, acc. numbers: KT756672.1, AY731737.1, MH878057.1
12/DD/4/07.03.16	*Candida parapsilosis*	100% identity to the *Candida parapsilosis*, acc. numbers: KP852497.1, FJ480839.1, MT176532.1
13/DD/5/07.03.16	*Candida parapsilosis*	100% identity to the *Candida parapsilosis*, acc. numbers: MT150862.1, MH545914.1, LC326042.1
14/DD/10/07.03.16	*Candida parapsilosis*	100% identity to the *Candida parapsilosis*, acc. numbers: KP852497.1, JQ965835.1, FJ746058.1
15/TW/8/05.04.16	*Clavispora lusitaniae*	100% identity to the *Clavispora lusitaniae*, acc. numbers: KT075270.1, KJ756765.1, KU728127.1
16/TW/10/05.04.16	*Candida parapsilosis*	99% identity to the *Candida parapsilosis*, acc. numbers: MT001266.1, MH545914.1, MK110314.1
17/RS/4/05.04.16	*Candida parapsilosis*	99% identity to the *Candida parapsilosis*, acc. numbers: KP852497.1, LC415311.1, MH481614.1
18/RS/5/05.04.16	*Candida parapsilosis*	100% identity to the *Candida parapsilosis*, acc. numbers: FJ480839.1, MT176532.1, LC413278.1
19/RS/10/05.04.16	*Candida parapsilosis*	99.5% identity to the *Candida parapsilosis*, acc. numbers: MH636034.1, JX441605.1, GU080054.1
20/DD/10/05.04.16	*Candida parapsilosis*	100% identity to the *Candida parapsilosis*, acc. numbers: MT151618.1, MH545914.1, MN545914.1
21/TW/10/11.05.16	*Candida parapsilosis*	100% identity to the *Candida parapsilosis*, acc. numbers: MT176532.1, MN067761.1, KU316730.1
22/RS/5/11.05.16	*Exophiala dermatitidis*	97% identity to the *Exophiala dermatitidis*, acc. numbers: MN447292.1, KF928510.1, MH878047.1
23/RS/8/11.05.16	*Clavispora lusitaniae*	99% identity to the *Clavispora lusitaniae*, acc. numbers: JQ665240, GQ179987.1, AJ539562.1
24/S/4/11.05.16	*Dipodascus capitatus*	100% identity to the *Dipodascus capitatus*, acc. numbers: KP761117.1, JF766627.1, KP761122.1
25/S/10/11.05.16	*Candida parapsilosis*	100% identity to the *Candida parapsilosis*, acc. numbers: KU316730.1, FJ480839, MT176532.1
26/DD/10/11.05.16	*Candida parapsilosis*	100% identity to the *Candida parapsilosis*, acc. numbers: KP852497.1, MH481614.1, KU316730.1
27/TW/5/13.06.16	*Candida parapsilosis*	100% identity to the *Candida parapsilosis*, acc. numbers: KP852497.1, MT176532.1, MH481614.1
28/TW/10/13.06.16	*Meyerozyma guilliermondii*	99% identity to the *Meyerozyma guilliermondii,* acc. numbers: MK907983.1, KY952484.1, KX907633.1
29/RS/4/13.06.16	*Candida parapsilosis*	100% identity to the *Candida parapsilosis*, acc. numbers: MT001266.1, MH545914.1, MK110314.1
30/RS/5/13.06.16	*Exophiala dermatitidis*	100% identity to the *Exophiala dermatitidis*, acc. numbers: MH876937.1, JN391398.1, KT756672.1
31/RS/10/13.06.16	*Meyerozyma guilliermondii*	100% identity to the *Meyerozyma guilliermondii,* acc. numbers: KMK034961.1, MH545918.1, MN653214.1
32/DD/1/13.06.16	*Candida parapsilosis*	100% identity to the *Candida parapsilosis*, acc. numbers: EF629545.1, MK110312.1, MT001266.1
33/DD/4/13.06.16	*Candida parapsilosis*	99% identity to the *Candida parapsilosis*, acc. numbers: FJ480839.1, MT176532.1, LC413278.1
34/DD/10/13.06.16	*Candida parapsilosis*	100% identity to the *Candida parapsilosis*, acc. numbers: MH613040.1, MT444982.1, MH612989.1
35/S/2/13.06.16	*Candida parapsilosis*	100% identity to the *Candida parapsilosis*, acc. numbers: MK110313.1, MH704191.1, MH545914.1

**Table 4:** Principal media used for the analysis of dishwashers and water for fungi (according to Ejdys et al. [63]).

Medium	Composition	
Sabouraud broth	Glucose	20 g
	Peptone	5 g
	Sodium chloride (NaCl)	2.5 g
	Distilled water	500 mL
	pH	7.2
Sabouraud agar with chloramphenicol	Glucose	20 g
	Peptone	5 g
	Agar	8 g
	Chloramphenicol	0.25 g
	Distilled water	500 mL
	pH	5.6–5.8
Slants with Sabouraud agar	Glucose	20 g
	Peptone	5 g
	Agar	11 g
	Distilled water	500 mL
	Glucose	2 g
	Galactose	2 g
	Saccharose	2 g
	Maltose	2 g
	Lactose	2 g
	Peptone K	0.5 g
Zymograms	0.1% Bromothymol blue solution in 95% ethyl alcohol	Few drops
	Aqueous solution of bromocresol purple	Few drops
	10% NaOH solution	Few drops
	Distilled water	100 mL
	pH	7.7
	Glucose	5 g
	Yeast-extract	0.5 g
	Dipotassium phosphate (K_2_HPO_4_)	2 g
	Monopotassium phosphate (KH_2_PO_4_)	1 g
Nickerson agar	Ammonium nitrate (NH_4_NO_3_)	0.5 g
	Sodium chloride (NaCl)	0.5 g
	Biotin	1.25 g
	Agar	7.5 g
	Distilled water	500 mL
	Trypan blue	0.05 g
